# Supplementary material for: End Sequence Analysis Toolkit (ESAT) expands the extractable information from single-cell RNA-seq data
Source: Genome Res. 2016 Oct;26(10):1397–410. doi: 10.1101/gr.207902.116 (PMC5052061; doi:10.1101/gr.207902.116)
Supplement: Supplemental Material [file supp_gr.207902.116_Supp_Materials.tar.gz › ESAT-esat_v0.1/doc/html/classumms_1_1core_1_1model_1_1score_1_1_poisson_enrichment_score-members.html]

ESAT: Member List


ESAT


- Main Page
- Related Pages
- Packages
- Classes
- Files

- Class List
- Class Index
- Class Hierarchy
- Class Members

All Classes Namespaces Files Functions Variables Enumerator Pages

- umms
- core
- model
- score
- PoissonEnrichmentScore

umms.core.model.score.PoissonEnrichmentScore Member List

This is the complete list of members for umms.core.model.score.PoissonEnrichmentScore, including all inherited members.

|  |  |  |
| --- | --- | --- |
| AbstractWindowScore(Annotation t) | umms.core.model.score.WindowScore.AbstractWindowScore |  |
| annotation | umms.core.model.score.WindowScore.AbstractWindowScore | protected |
| asRPKM(double count, double total, int windowSize) | umms.core.model.score.CountScore | static |
| calculatePVal(double a, double b, double lambdaSample, double lambdaCtrl, double w) | umms.core.model.score.PoissonEnrichmentScore |  |
| compareTo(CountScore o) | umms.core.model.score.CountScore |  |
| CountScore(Annotation a) | umms.core.model.score.CountScore |  |
| CountScore(CountScore other) | umms.core.model.score.CountScore |  |
| CountScore(CountScore other, Annotation newAnnotation, double newCount) | umms.core.model.score.CountScore |  |
| CountScore(AnnotationCollection<?extends Annotation > model, Annotation annotation) | umms.core.model.score.CountScore |  |
| CountScore(AnnotationCollection<?extends Annotation > model, Annotation annotation, double regionTotal) | umms.core.model.score.CountScore |  |
| CountScore(AnnotationCollection<?extends Annotation > model, Annotation annotation, boolean fullyContained) | umms.core.model.score.CountScore |  |
| CountScore(AnnotationCollection<?extends Annotation > model, Annotation annotation, double regionTotal, boolean fullyContained) | umms.core.model.score.CountScore |  |
| CountScore(Annotation annotation, double count, double regionTotal, double total) | umms.core.model.score.CountScore |  |
| DEFAULT\_REGION\_TOTAL | umms.core.model.score.PoissonEnrichmentScore | static |
| equals(Object o) | umms.core.model.score.CountScore |  |
| getAnnotation() | umms.core.model.score.WindowScore.AbstractWindowScore |  |
| getAverageCoverage(AlignmentModel data, CoordinateSpace coordSpace) | umms.core.model.score.PoissonEnrichmentScore |  |
| getCount() | umms.core.model.score.CountScore |  |
| getCtrlCount() | umms.core.model.score.PoissonEnrichmentScore |  |
| getCtrlLambda() | umms.core.model.score.PoissonEnrichmentScore |  |
| getCtrlRegionTotal() | umms.core.model.score.PoissonEnrichmentScore |  |
| getCtrlSampleLambda() | umms.core.model.score.PoissonEnrichmentScore |  |
| getCtrlTotal() | umms.core.model.score.PoissonEnrichmentScore |  |
| getGlobalLength() | umms.core.model.score.PoissonEnrichmentScore |  |
| getLocalSampleLambda() | umms.core.model.score.PoissonEnrichmentScore |  |
| getPvalue() | umms.core.model.score.PoissonEnrichmentScore |  |
| getRegionLength() | umms.core.model.score.PoissonEnrichmentScore |  |
| getRegionTotal() | umms.core.model.score.CountScore |  |
| getRPK() | umms.core.model.score.CountScore |  |
| getRPKM() | umms.core.model.score.CountScore |  |
| getSampleCount() | umms.core.model.score.PoissonEnrichmentScore |  |
| getSampleLambda() | umms.core.model.score.PoissonEnrichmentScore |  |
| getSampleRegionTotal() | umms.core.model.score.PoissonEnrichmentScore |  |
| getSampleTotal() | umms.core.model.score.PoissonEnrichmentScore |  |
| getScore() | umms.core.model.score.CountScore |  |
| getTotal() | umms.core.model.score.CountScore |  |
| hashCode() | umms.core.model.score.CountScore |  |
| PoissonEnrichmentScore(AlignmentModel sample, AlignmentModel ctrl, Annotation a) | umms.core.model.score.PoissonEnrichmentScore |  |
| PoissonEnrichmentScore(AlignmentModel sample, Annotation a) | umms.core.model.score.PoissonEnrichmentScore |  |
| refreshPvalue() | umms.core.model.score.CountScore |  |
| setCount(double count) | umms.core.model.score.CountScore |  |
| setCtrlCount(double d) | umms.core.model.score.PoissonEnrichmentScore |  |
| setCtrlRegionTotal(double d) | umms.core.model.score.PoissonEnrichmentScore |  |
| setCtrlTotal(double d) | umms.core.model.score.PoissonEnrichmentScore |  |
| setGlobalLength(double d) | umms.core.model.score.PoissonEnrichmentScore |  |
| setPvalue(double scanPvalue) | umms.core.model.score.PoissonEnrichmentScore |  |
| setRegionLength(double regionLength) | umms.core.model.score.PoissonEnrichmentScore |  |
| setRegionTotal(double regionTotal) | umms.core.model.score.CountScore |  |
| setSampleCount(double d) | umms.core.model.score.PoissonEnrichmentScore |  |
| setSampleRegionTotal(double d) | umms.core.model.score.PoissonEnrichmentScore |  |
| setSampleTotal(double d) | umms.core.model.score.PoissonEnrichmentScore |  |
| setTotal(double total) | umms.core.model.score.CountScore |  |
| toString() | umms.core.model.score.CountScore |  |


---

Generated on Fri Aug 15 2014 15:11:13 for ESAT by  

 1.8.7
